# The “red herring” after 20 years: ageing and health care expenditures

**DOI:** 10.1007/s10198-020-01203-x

**Published:** 2020-06-04

**Authors:** Friedrich Breyer, Normann Lorenz

**Affiliations:** 1 Department of Economics, University of Konstanz, P.O. Box 135, 78457 Konstanz, Germany; 2 University of Trier, Universitätsring 15, 54296 Trier, Germany

**Keywords:** H51, J14, I10

## Introduction

One of the most important controversies in the health economics discourse of the last twenty years concerns the question whether the imminent ageing of the population in most OECD countries will place an additional burden on the tax-payers who finance public health care systems. These systems are usually pay-as-you-go financed with taxes or contributions depending on labor income and pensions. Population ageing due to rising longevity and below-replacement fertility in coming decades will lower the population share of working-age persons and raise the share of pensioners. Since labor income exceeds pensions by far, this will weaken the tax base so that tax or contribution rates will rise notably.

In addition and as suggested by cross-sectional data, if the elderly had higher health care expenditures (HCE) than the young, the trend of rising contribution rates would be reinforced. Considering that other branches of social insurance (notably pensions and long-term care) are also unfunded and involve growing transfers from working-age to retired people in the process of population ageing, the social insurance system as a whole may become unsustainable in the near future.

The view that per-capita HCE are rising because of population ageing has been questioned on methodological grounds in a path-breaking article by Zweifel, Felder and Meier [31], who attacked the practice of relying on a cross-sectional correlation when forecasting future HCE. In fact, they dubbed the observed correlation between a person’s age and his or her HCE a “red herring” (i.e., a false clue) because most of this correlation is due to the fact that HCE rise steeply in the last months before death and at higher ages more and more persons are in their last year of life. Thus, population ageing due to rising longevity would simply shift the high death-related costs to higher ages.

In the 20 years since the publication of the Zweifel et al. paper, a large literature has emerged in which the “red herring hypothesis” (RHH) has been tested using different datasets, different empirical approaches and more refined methods. Furthermore, the implications of these findings for the forecast of future HCE have been examined in a number of papers. However, despite the ink spilled on the topic, it is not even entirely clear what the RHH precisely means. Therefore, we start in the following section with the statement of four different versions of the RHH and the logical relationships among them. After that, a critical review of the empirical tests of (the different versions of) the RHH is provided, then we summarize the literature on the implications for HCE forecasts and a further section is devoted to a discussion of policy implications, before we conclude.

## The red herring hypothesis: theory

There is not a unique RHH, but in fact four different versions of it in the literature, which we shall call RHH-1 to RHH-4:

RHH-1: Population ageing due to rising life expectancy as such does not cause an increase in per-capita HCE [31].^1^

RHH-2: The increase in per-capita HCE with age in descriptive data is partly (or even predominantly) due to the fact that in older age groups more individuals are in their last year(s) of life in which HCE are particularly high [14]. This is also known as the “time-to-death hypothesis”.

RHH-3: In a regression equation for individual HCE, the age variable(s) become(s) weak or zero, once time-to-death (TTD) is included [19, 31].

RHH-4: In a regression equation for individual HCE, the estimated age gradient becomes much smaller, when the analysis distinguishes between decedents (persons in their last *x* years of life, where *x* is a small number, often three or four) and survivors (all the others) [32]. In the following we will interpret it as a confirmation of this hypothesis if one of the age gradients becomes negative.

The main difference between these four statements is that RHH-2 to RHH-4 refer to cross-sections of individual data, whereas RHH-1 is a claim about the behavior of aggregate HCE in a country over time. Thus it is necessary to examine the logical relations between them, and in particular to answer the question which of the last three hypotheses, if empirically confirmed, implies that RHH-1 is true.

1. On a general level, a claim on a dynamic development of HCE does not follow directly from any of the statements on cross-section data. First, the cross-sectional relationship between age and HCE does not have to be stable as time progresses and population ages. E.g., empirical studies [13] have shown that the age gradient of HCE has steepened over time in some countries. Secondly, an exogenous increase in life expectancy over time may have an independent impact on per-capita HCE, as proposed by Breyer et al. [7] and dubbed “Eubie-Blake effect”. When physicians consider performing a complicated, expensive or risky treatment (such as hip replacement) on an elderly patient, they will deem it the more worthwhile the longer they think the patient will benefit from the treatment. Thus, the higher the (estimated) remaining life expectancy of the patient, the more of these expensive or risky treatments will be performed and thus the higher will be per-capita HCE.
  But even apart from this general problem of mixing static and dynamic properties, RHH-1 does not follow from any of the other three hypotheses, even if they were confirmed:
2. RHH-4 implies RHH-2, but neither of the two implies RHH-1: If the age gradient decreases when determined separately for survivors and decedents, part of the “raw” age gradient is due to the “high costs of dying”, so RHH-2 is confirmed. However, this does not mean that ageing does not raise HCE as long as the age gradient for decedents is still positive. Even if it is negative, but the one for survivors is large enough, an increase in the share of the elderly may suffice to raise per-capita HCE as we explain in greater detail in 3 below.
3. Existing empirical confirmations of RHH-3 do not imply RHH-1, either. Notice first that in descriptive individual data, the relationship between age and HCE for decedents is negative beyond a certain age (usually about 50 years),^2^ and positive in this age group for survivors. Moreover, per-capita HCE for decedents exceed the highest HCE for survivors at any age (the TTD-effect). Now for reasons of data availability, TTD can be observed mainly for persons for which this variable is relatively small, i.e. for decedents [31] use 2–5 years, [19] up to 7 years). Both papers, which deal exclusively with decedents, find no significant effect of age on HCE and thus a zero age gradient for decedents, but others have confirmed the negative age gradient found in descriptive data. Combining the zero or negative age gradient with the positive age gradient for survivors, an increase in life expectancy has three effects on per-capita HCE: (1) average HCE in the survivor category rises, (2) the share of survivors increases, and (3) average HCE in the decedent category stays constant or decreases. The first effect on HCE is positive, the second is negative from the TTD effect, and the third effect is zero or negative. Thus the total effect is ambiguous. Moreover, the key argument brought forward in the famous “red herring” paper by Zweifel et al. [31] that *because the third effect is zero*, the total effect is not positive, is a non-sequitur. On the contrary, a negative age gradient of HCE among decedents would have made the validity of RHH1 more likely.

## Empirical tests of the red herring hypothesis

While the following survey is necessarily incomplete, we focus on those papers who have played a key role in the historical development of the debate on the Red Herring Hypothesis.

### Individual data

The seminal paper by Zweifel et al. [31] is based on the analysis of HCE of approximately 1000 members of two Swiss sickness funds who died between 1983 and 1992, most of them at the age above 65 years. The expenditure data are quarterly total HCE and are available for the last eight quarters before death. The authors found that HCE were significantly and steeply rising towards the individual’s death, while the patient’s age turned out to be insignificant, even when the time period before death was extended to 20 quarters. They concluded: “Exclusive emphasis on population ageing as a cause of growth in per capita health care expenditure runs the risk of creating a red herring by distracting from the choices that ought to be made …” (p. 494) and immediately above this sentence: “attention is diverted from the real causes of growth of the health care sector, which are failures in insurance markets, technical progress in providing health care services, and wrong incentives for patients, doctors and hospitals caused by government regulation of the health care sector.”

The paper not only convincingly confirms the TTD hypothesis, but also marks the birth of the red-herring claim which gave its name to a whole branch of the health economics literature.^3^ The quotation shows that the authors suggest that the apparent relationship between population ageing and HCE was created by politicians and health care officials who wanted to distract public attention from the real reasons for the observed increase in HCE: inefficiency and overprovision of health care services. However, as their empirical analysis confirms RHH-3 (which, as shown above, contradicts RHH-1), this conclusion cannot be drawn.

Zweifel et al. [32] again examined Swiss health insurance data referring to the year 1999, but this time for both groups, decedents and survivors. Estimating a two-part model, they find that the increase of predicted HCE with age beyond age 50 in each of these groups is considerably smaller than the one in the descriptive data of the whole group. This confirms RHH-4 (but not RHH-3). However, the specification of the regression equation can be criticized on two accounts: first, the age of the patient is measured only by age and age-squared, forcing a parabolic relationship between age and HCE. One of the problems of this approach, which the authors acknowledge, is that it does not consider more complicated functional relationships: in particular, the convex shape of HCE between ages 40 and 70 and the decrease with age at very high ages, can not both be captured by a parabola. Secondly, the estimation was not performed separately for survivors and decedents, but in a single regression equation in which the survival status was accounted for only by a binary variable, and interacted with the linear age variable, but not with age-squared. As decedents account for only about four per cent of all individuals in the data set, this implies that the shape of the parabola is mainly determined by the U-shaped age-expenditure profile of survivors. Thus, it appears that HCE in the last year(s) of life are lowest at about age 50 and increase more than proportionally with age thereafter – which is exactly the opposite shape to the one observed in descriptive data and in other studies.

Shang and Goldman [27] used a 2SLS procedure to first explain TTD (which they call „life expectancy “) and then HCE. They confirm the original Zweifel et al. result that “age has little additional predictive power on health care expenditures after controlling for life expectancy” (p.487), but then add that “the predictive power of life expectancy itself diminishes as health status measures are introduced into the model” (ibid.). In a way, this amounts to throwing the baby out with the bath water since, first, it is trivial that HCE depend on the health of the patient and, secondly, when one wants to predict the future course of HCE in a country, while information on the age structure of the population is often available, forecasts of the prevalence of specific diseases such as diabetes, cancer or stroke are typically not. This begs the question how this particular finding can be used.

Several studies have focussed on expenditures for specific types of services such as hospital care, outpatient care or long-term care (LTC). Although such an approach is of limited value when one is interested in the question whether population ageing constitutes a threat to the financial sustainability of public health care systems, in some cases this choice is inevitable for reasons of data availability. Seshamani and Gray [26], e.g., showed that hospital costs in Britain started rising as early as 15 years before a patient’s death (and increased by a factor of ten from 5 years before death until the last year), whereas the increase of the costs of the final year with age between 65 and the peak at 80 amounted to only about 30 per cent. Wong et al. [30] examined disease-specific hospital expenditures in the Netherlands for 94 different conditions and found that there was a clear effect of proximity of death on HCE, while the impact of age was relatively modest.

In contrast to the results on hospital expenditures, Atella und Conti [2], who examined a large sample of 750,000 adult persons in Italy in the time period 2006–2009, found for primary care expenditures (pharmaceuticals, diagnostic tests and specialist visits) that age is a much better predictor than TTD: while expenditures increased fivefold between age 40 and 80, the increase in the last year of life amounted to only 30 per cent compared to an average year. A significant impact of age and a much lesser impact of TTD could also be established for long-term care (LTC) expenditures by De Meijer et al. [11] for the Netherlands and Karlsson and Klohn [22] for Sweden.

### Country data

An important weakness of most of the empirical analyses discussed above is their dependence on cross-sectional data so that the impact of population ageing due to rising longevity, which is a development over time, could not be measured directly. To deal with this problem, it is necessary to analyze time series or panel data. In some of these studies so far the units of observation are not individuals, but populations of whole countries.

Several studies did not reveal a significant effect of the share of the elderly on per capita HCE of a country. Examples are Getzen [16] for a panel of 20 OECD countries over 28 years (1960–88) and Barros [4] for a panel of 24 OECD countries over three decades (1960–90). In contrast, Zweifel et al. [33], who examined the mutual dependence of longevity and HCE, found for a panel of 30 OECD countries over three decades (1970–2000) that the variable which measures both life expectancy and the share of citizens of age 65 + was a significant positive determinant of per-capita HCE. In a recent study for Switzerland, Colombier [9] showed that real per-capita GDP, the old-age dependency ratio and R&D expenditures in the US health care sector (as a proxy for medical innovation) significantly increased per-capita HCE, whereas a positive and significant impact of the mortality rate could not be identified.

## Implications for future expenditure increases

The authors of the studies discussed above aimed at uncovering the relationship between calendar age or population ageing and HCE in the past. The main target of this literature is to make empirically well-founded predictions on the future development of expenditures for health care and LTC. Such endeavors are to some degree problematic, as they can not take political decisions into account which might be taken in view of the scarcity of public funds. The simulations should thus be interpreted as providing estimates of future expenditures under the same rationing rule (or development of the rationing rule over time) as existed in the past.

The most important conclusion from studies in this branch of the literature is that simulations on the basis of empirical estimates predict a less dramatic increase in future expenditures when the variable TTD is included among the regressors than otherwise. This was first shown by Stearns and Norton [28] for the US. They compared forecasts of Medicare expenditures in the year 2020 based on expenditure data of the period 1992–1998 and found that ignoring the TTD effect leads to an overestimate of total HCE of a Medicare recipient (starting from age 65) by up to 15 per cent (namely to 117,000 instead of 102,000 USD). Compared to the starting level of 53,000 USD in 1990, the extent of overestimation amounted to almost one-third of the „true“ growth. Other studies for various countries found different values for the forecast error. On the low end, Van Baal and Wong [29] for the Netherlands and Colombier and Weber [10] for Switzerland found no effect of death-related costs on long-run simulations of HCE in their countries, Polder et al. [25] calculated for the Netherlands an overestimate of the HCE growth rate due to the „wrong” model of 10 per cent, and Geue et al. [17] found an overestimate of about 17 per cent for hospital expenditures in Scotland. A considerably stronger overestimation effect of about 50 per cent was found by Bjørner and Arnberg [5] for Denmark. Moreover, several studies (such as Breyer and Felder [6] for Germany and Dormont et al. [12] for France) found that although the effect of population ageing on HCE is positive, the impact of medical progress is considerably larger.

## Discussion and policy implications

This review shows that the findings on the relationship between ageing and HCE are contradictory and therefore inconclusive. This is partly due to the use of different empirical strategies. In particular, while there is solid support for hypotheses RHH-2 (the TTD hypothesis) and RHH-4 (the importance of distinguishing between “survivors” and “decedents”), before any definite conclusion on the policy relevant hypothesis RHH-1 can be drawn, a number of methodological issues have to be resolved:

1. As the functional relationship between age and individual HCE is not simply parabolic, finer measures than age and age squared, such as 5-year age brackets or semiparametric methods are recommended. The former are used by Karlsson et al. [20] on data from German private health insurance and the latter by [24] with data from German SHI.
2. When distinguishing between survivors and decedents a decision has to be made for how many years before death a person is regarded a decedent. Ideally, this dividing line should be drawn according to the criterion how long before its occurrence imminent death has an influence on HCE and thus should depend upon the cause of a person’s death. For people dying from cancer, e.g., the time span between the onset of the disease and death varies greatly by the type of cancer. In contrast, victims of fatal accidents never reach the status of a decedent for a time span worth mentioning. Thus the decision will typically be made for pragmatic reasons: each additional year before death that is shifted into the decedent category implies the loss of one year of data in the survivor category. In existing studies with this distinction the time span before death is usually defined between 3 and 4 years.
3. A related issue concerns studies based on “pure” decedent data such as Zweifel et al. [31] and Howdon and Rice [19]. This approach cannot yield reliable insights on the overall problem of ageing and HCE because only between 12 and 16 per cent of all HCE is incurred in the last 3 years before death (see, e.g., Bakx [3] or Karlsson et al. [21].
4. An extremely important question is how the observed expenditure data have to be interpreted: Do they constitute demand or even medical “need” or are they the result of rationing? Whenever it is the purpose of the studies to assess the future sustainability of health care financing systems, it seems desirable to forecast future “need” however this is defined. On this account, cross-sectional studies based on individual data appear to be most appropriate because relative differences in individual expenditures within a specific health system at a point in time most likely reflect differences in medical necessity (at least in systems with universal access and without important patient cost-sharing). In contrast, results from aggregate data tend to depend most heavily on rationing rules prevailing in the corresponding countries. It is therefore not surprising that these studies failed to uncover any effect of the age structure of the population on per-capita HCE. Assuming that the rationing rule is based on some kind of national health care budget, this fixes the expenditures and their growth rate, no matter how the share of the elderly population changes from one year to the next.
5. A related question with respect to RHH-1 is through what channel population ageing should lead to rising per-capita HCE. The bulk of the literature seems to take it for granted that the channel is medical need: as morbidity rises with age (and ignoring a TTD effect), a larger share of older people should lead to higher HCE. But the channel can also be political pressure of the electorate: with a higher share of elderly voters, there is more political demand for public HCE, and more public HCE in turn will result in increased longevity and therefore a higher share of the elderly in the electorate, who will vote for higher public HCE, and so on. Thus, there may be a vicious circle or, as the originators of this hypothesis called it, a “Sisyphus Syndrome” in health care, which was empirically confirmed by Zweifel et al. [33]. Taking this result for granted, it is no longer the question *whether* ageing raises HCE, but only *how* this comes about.
6. The findings of this paper on the negative impact of lagged HCE on mortality call the assumption of the exogeneity of the ageing variables into question. Indeed, there is a large literature on the impact of health care spending on health outcomes including mortality and life expectancy (see, e.g., [1] and, for a meta-regression, [15]). For space limitations, we cannot elaborate further on this point.
7. Furthermore, it is crucial what types of expenditures are included in the analysis: those for medical services in the narrower sense or also those for LTC. It appears that a positive association between ageing and HCE is more prevalent for LTC than for other health expenditures. From the perspective of the compression of morbidity hypothesis, this is surprising: if the total increase in longevity consists in “healthy” years and if LTC need is always caused by illness, then LTC expenditures should only depend on TTD and not at all on age. As a consequence, age-specific LTC shares should fall when life expectancy rises. However, this cannot be observed in German LTC insurance data.^4^
8. When comparing the size of the ageing effect with other causes of HCE growth, it is important to properly identify the “time trend”, which plays a large role in many time-series or panel studies. It is obvious that it captures all time-varying factors which are not explicitly accounted for in the regression. Some authors interpret it as the effect of technological change [8], p.6) or medical progress, but part of it can also be due to other time-varying factors such as GDP. When income grows, people might be willing to spend more on health, and they can do so without any changes in the tax or contribution rate as long as expenditures grow in line with GDP. In countries with a strong tradition of stable rates of (payroll) taxes for financing Social Health Insurance it is the difference between the growth rates of GDP and HCE that needs an explanation. Part of it may be given by medical progress, another part by “Baumol’s cost disease” [18], which states that unit costs increase in labor-intensive sectors, in which the productivity growth falls short of the general growth of wages.
9. Finally, even if it was true that population ageing had a positive, but small effect on HCE in the past, this would not necessarily imply that this effect must remain small in the future. For whether an exogenous variable *x* has a large impact on an endogenous variable *y* depends on the extent to which *x* varies, and the speed at which populations are ageing will increase considerably in the coming decades. Looking at the development of the old-age dependency ratio OADR (number of persons over 65 as a percentage of the population between 20 and 64) in the OECD, it is obvious that in the last 30 years of the twentieth century, this ratio increased only by a few percentage points so that at the time of writing, Zweifel et al. [31] were safe in stating that population ageing could not have been a major cause of HCE growth until that time.^5^ In contrast, the coming decades will see much bigger increases in the OADR, which amount to 20 or more percentage points in many countries and to over 30 percentage points in Southern Europe. Therefore, even if the effect of a one-percentage-point increase of the OADR on average HCE is a small number, multiplying this number by 30 may yield a sizeable overall effect.

Although no final verdict on the validity of RHH-1 is possible at this point, it is an independent question whether the ageing-HCE relationship is truly a “red herring” in political debates on health care reforms – in the sense that it “detracts from the choices that ought to be made”. Ironically, this assertion can easily be turned around: if politicians know that the public health insurance system is unsustainable, they might—one would hope—take measures to prevent the most negative consequences of a breakdown of the system, e.g. by curtailing less cost-effective services from its benefit package and encourage prevention. Now, the publication and dissemination of the early red herring literature, in particular of the claim RHH-1 may lead journalists and politicians to believe that the imminent ageing of the population—which will be far more severe than in the past (see item 9)—will not cause any problems for the financial viability of social health insurance. Thus it is the red herring hypothesis itself that carries with it the danger to “detract from the choices that ought to be made”.

## Concluding remarks

A tentative conclusion that can be drawn from this survey is that future population ageing will have a positive impact on HCE, but the size of this impact depends on the type of service, with a larger one for LTC than for acute care. Moreover, it can be argued that the growth rate that is caused by population ageing is small compared to the one which is due to other time-varying factors such as medical progress and rising GDP. On the other hand, as long as the growth rate does not exceed GDP growth, it does not jeopardize the financial viability of publicly financed health care systems provided that tax bases do not become more volatile over time. Thus, when GDP growth is deducted from the time trend, then it is no longer clear that the impact of population ageing is “small” relative to this difference.

In any case, there is still room for improvement in the methodology of measuring the impact of population ageing on HCE. As data availability and quality improves, more precise measures will hopefully be possible.

## Footnotes

1 „…per capita HCE is not necessarily affected by the ageing of the population due to an increase in life expectancy. Rather, an increase in the elderly’s share of population seems to shift the bulk of HCE to higher age, leaving per capita HCE unchanged” (p. 493).

2 See, e.g., Levinsky et al. [23] for the U.S.

3 Readers of criminal stories know that a „red herring” is a false clue that has been deliberately laid by the culprit (or the author) to lead the detective (or the reader) astray.

4 See Gesundheitsberichterstattung des Bundes (www.gbe-bund.de), Gesundheitliche Lage/Krankheitsfolgen/Pflegebedürftigkeit, Pflegebedürftige (Anzahl und Quote).

5 Source: United Nations, Department of Economic and Social Affairs, World Population Prospects 2019.

## References

[1] Aisa R, Clemente J, Pueyo F (2014). The influence of (public) health expenditure on longevity. Int. J. Public Health.

[2] Atella V, Conti V (2014). The effect of age and time to death on primary care costs: the Italian experience. Soc. Sci. Med..

[3] Bakx P, O’Donnell O, van Doorslaer E (2016). Spending on Health Care in the Netherlands: not going so Dutch. Fiscal Studies.

[4] Barros PP (1998). The black box of health care expenditure growth determinants. Health Econ..

[5] Bjørner TB, Arnberg S (2012). Terminal costs, improved life expectancy and future public health expenditure. Int. J. Health Care Finance Econ..

[6] Breyer F, Felder S (2006). Life expectancy and health care expenditures: a new calculation for Germany using the costs of dying. Health Policy.

[7] Breyer F, Lorenz N, Niebel T (2015). Population ageing and health care expenditures: Is there a Eubie Blake Effect?. Eur. J. Health Econ..

[8] Chernew, M.E. and J.P. Newhouse (2012), Health care spending growth. In: P.P Barros and T. McGuire, eds., *Handbook of Health Economics, vol.2*, Amsterdam, pp 1–43.

[9] Colombier C (2018). Population ageing in healthcare – a minor issue? Evidence from Switzerland. Appl. Econ..

[10] Colombier C, Weber W (2011). Projecting health-care expenditure for Switzerland: further evidence against the “red herring” hypothesis. Int. J. Health Plan. Manag..

[11] De Meijer C, Koopmanschap M, Bago d’Uva T, van Doorslaer E (2011). Determinants of long-term care spending: age, time to death or disability?. J. Health Econ..

[12] Dormont B, Grignon M, Huber H (2006). Health expenditure growth: reassessing the threat of ageing. Health Econ..

[13] Felder S, Werblow A (2008). Does the age profile of health care expenditure really steepen over time? New evidence from Swiss cantons. Geneva Papers Risk Insurance.

[14] Fuchs VR (1984). Though much is taken: reflections on aging, health and medical care. Milbank Memorial Fund Q. Health Soc..

[15] Gallet CA, Doucouliagos H (2017). The impact of healthcare spending on health outcomes: a metaregression analysis. Soc. Sci. Med..

[16] Getzen TE (1992). Population aging and the growth of health expenditures. J. Gerontol..

[17] Geue C, Briggs A, Lewsey J, Lorgelly P (2014). Population ageing and healthcare expenditure projections: new evidence from a time to death approach. Eur. J. Health Econ..

[18] Hartwig J (2008). What drives health care expenditure?—Baumol’s model of ‘unbalanced growth’ revisited. J. Health Econ..

[19] Howdon D, Rice N (2018). Health care expenditures, age, proximity to death and morbidity: implications for an ageing population. J. Health Econ..

[20] Karlsson M, Iversen T, Öien H (2018). Aging and healthcare costs. Oxf. Res. Encyclopedia Econ. Fin..

[21] Karlsson M, Klein TJ, Ziebarth N (2016). Skewed, persistent and high before death: medical spending in Germany. Fiscal Stud..

[22] Karlsson M, Klohn F (2014). Testing the red herring hypothesis on an aggregated level: ageing, time-to-death and care costs for older people in Sweden. Eur. J. Health Econ..

[23] Levinsky NG, Yu W, Ash A, Moskowitz M, Gazelle G, Saynina O, Emanuel EJ (2001). Influence of age on Medicare expenditures and medical care in the last year of life. JAMA.

[24] Lorenz, N., Ihle, P., Breyer, F.: Aging and health care expenditures: a non-parametric approach. CESifo Paper 8216 (2020)

[25] Polder JJ, Barendregt JJ, van Oers H (2006). Health care costs in the last year of life – the Dutch experience. Soc. Sci. Med..

[26] Seshamani M, Gray A (2004). A longitudinal study of the effects of age and time to death on hospital costs. J. Health Econ..

[27] Shang B, Goldman D (2008). Does age or life expectancy better predict health care expenditures?. Health Econ..

[28] Stearns SC, Norton EC (2004). Time to include time to death? The future of health care expenditure predictions. Health Econ..

[29] Van Baal PH, Wong A (2012). Time to death and the forecasting of macro-level health care expenditures: some further considerations. J. Health Econ..

[30] Wong A, van Baal PHM, Boshuizen HC, Polder JJ (2011). Exploring the influence of proximity to death on disease-specific hospital expenditures: a Carpaccio of red herrings. Health Econ..

[31] Zweifel P, Felder S, Meier M (1999). Ageing of population and health care expenditure: a red herring?. Health Econ..

[32] Zweifel P, Felder S, Werblow A (2004). Population ageing and health care expenditure: new evidence on the "red herrings". Geneva Papers Risk Insurance.

[33] Zweifel P, Steinmann L, Eugster P (2005). The Sisyphus syndrome in health revisited. Int. J. Health Care Econ. Financ..

